# Investigation of AKI with Early Biomarkers After Cardiac Surgery

**DOI:** 10.21470/1678-9741-2019-0178

**Published:** 2020

**Authors:** Hüseyin Mutlu, Emel Gündüz, Tülin Aydoğdu Titiz, İkbal Özen Küçükçetin

**Affiliations:** 1 Department of Anesthesiology and Reanimation, Faculty of Medicine, Akdeniz University, Antalya, Turkey.

**Keywords:** Lipocalin-2, CST3 protein, human, Cystatin C, Interleukin-18, Risk Factors, Acute Kidney Injury, Cardiac Surgical Procedures, Biomarkers, Kidney, Postoperative Complications

## Abstract

**Objective:**

To provide a new interpretation of the effect of intraoperative hemodynamic data on postoperative acute kidney injury (AKI) development and to determine the accuracy of some biomarkers which are thought to be the early markers of renal injury.

**Methods:**

One hundred adult patients who were connected to the heart-lung pump during open-heart surgery were included in this study. Hemodynamic data, oxygen delivery, and transfusions were recorded intraoperatively, and the preoperative and 3. postoperative hour cystatin C, interleukin-18 (IL-18), and neutrophil gelatinase-associated lipocalin (NGAL) parameters were measured for early detection of kidney damage. In the analysis, 95% significance level was used to determine the difference.

**Results:**

According to the Kidney Disease Improving Global Outcomes criterion, AKI developed in 24 patients, 18 of whom were stage 1, two were stage 2, and four were stage 3. AKI (+) patients had more transfusions in the intraoperative period and AKI development was a risk factor for postoperative complications. NGAL and IL-18 levels were found to be approximately two-fold in the postoperative period in AKI (+) patients, whereas cystatin C was not sensitive in AKI detection.

**Conclusion:**

AKI development increases the risk of postoperative complications. NGAL and IL-18 were successful in detecting AKI in the early postoperative period.

**Table:** 

Abbreviations, acronyms & symbols				
ADH	= Antidiuretic hormone		HLP	= Heart-lung pump
AKI	= Acute kidney injury		IL-18	= Interleukin-18
AKIN	= Acute Kidney Injury Network		KDIGO	= Kidney Disease Improving Global Outcomes
BMI	= Body mass index		LVAD	= Left ventricular assist device
CABG	= Coronary artery bypass grafting		MAP	= Mean arterial pressure
CAP	= Central arterial pressure		MPP	= Mean perfusion pressure
COPD	= Chronic obstructive pulmonary disease		NGAL	= Neutrophil gelatinase-associated lipocalin
CPB	= Cardiopulmonary bypass		PAP	= Pulmonary artery pressure
CSA-AKI	= Cardiac surgery-associated acute kidney injury		RIFLE	= Risk, Injury, Failure, Loss of function, and End-stage kidney disease
CVP	= Central venous pressure		RRT	= Renal replacement therapy
DO_2_	= Oxygen delivery		SS	= Standard deviation values
DPP	= Diastolic perfusion pressure			
FFP	= Fresh frozen plasma			

## INTRODUCTION

Cardiac surgery-associated acute kidney injury (CSA-AKI), which is one of the important complications that develop after cardiac surgery, is seen in approximately 30% of the cases and is the second most common cause of postoperative acute kidney injury (AKI)^[1]^.

AKI is associated with increased morbidity, mortality, and prolonged hospital stay. Thirty-day mortality increases three times even in patients with a minimal increase in serum creatinine levels after cardiac surgery, and mortality increases by 63% in patients with severe AKI requiring renal replacement therapy (RRT)^[2]^.

In the recent years, the first consensus criterion published for AKI identification was the RIFLE (acronym for Risk, Injury, Failure, Loss of function, and End-stage kidney disease) and it was revised in 2007 by the Acute Kidney Injury Network (AKIN). In 2012, a new definition was introduced by the Kidney Disease Improving Global Outcomes (KDIGO) guidelines^[3]^.

The incidence of CSA-AKI varies according to the identification criterion. It is between 9% and 39% according to RIFLE and AKIN criteria. According to the KDIGO criterion introduced in the last decade, this rate varies between 3.1% and 42%^[3]^.

Many factors play a role in CSA-AKI etiopathogenesis. These include endogenous and exogenous toxins, ischemia-reperfusion injury, neurohormonal activation, inflammation, oxidative stress, etc. These associated factors often coexist in the perioperative period, instead of occurring separately, increasing the risk of injury. There are also factors associated with the patient contributing to perioperative kidney injury.

Measurement of renal function is classically evaluated based on serum creatinine levels and urine output. Literature data on some biomarkers recommended for early detection of renal damage due to the limitations of these two parameters are contradictory. Among all recommended biomarkers, the markers which are reported to be more sensitive than others in detecting AKI are neutrophil gelatinase-associated lipocalin (NGAL) and interleukin-18 (IL-18)^[3]^.

In this study, we aimed to provide new insight into the contribution of intraoperative hemodynamic data to postoperative AKI in patients who were connected to the heart-lung pump (HLP) during open-heart surgery and to determine the accuracy of some biomarkers which are thought to be early markers of kidney injury.

## METHODS

One hundred patients at the age of 18 years and over, who were connected to HLP during open-heart surgery between January 2018 and June 2018 at Akdeniz University Faculty of Medicine Hospital, were included in the study. Ethical committee approvals and patient consent were obtained before the study. This research was funded by the Akdeniz University Scientific Research Projects - SRP board.

Preoperative demographic data, comorbidities, and preoperative laboratory parameters of the patients included in the study were recorded. Haemodynamic data of patients undergoing open-heart surgery - measured and calculated intraoperatively - were recorded and fluid treatments, transfusions, and urine volume during this period and systemic oxygen presentations during the cardiopulmonary bypass period were calculated.

Patients were followed up until the 7. postoperative day, and AKI diagnosis was based on KDIGO criterion.

Biomarkers which are thought to be early markers of kidney damage were studied by the enzyme-linked immunosorbent assay method on blood samples taken before anesthesia induction in the preoperative period and blood samples taken at the 3. postoperative hour.

### Statistical Analysis

Student's *t*-test was used for the analysis of binary variables and chi-square significance test was used for the analysis of categorical variables. Mean values of continuous variables were analyzed by repeated measures analysis of variance. A 95% significance level (or α = 0.05 error margin) was used to determine statistically significant differences in the analyses.

Biochemical data were analyzed using the IBM SPSS Statistics 18© Copyright SPSS Inc. 1989, 2010 software and the GraphPad Prism 5 software.

## RESULTS

Of the 100 patients included in the study, 29 were female and 71 were male. Demographic data of the patients are shown in Table 1.

**Table 1:** Demographic data of all patients included in the study.

		Min-max	Median	Mean ± SS N%	
Age		24.0-84.0	60.5	64.52±9.32	
Gender	Female			29	29%
	Male			71	71%
Weight (kg)		52-135	76.5	77.33±13.2	
BMI (kg/m2)		19.8-41.2	26.58	27.10±4.3	
Diabetes mellitus				29	29%
Hypertension				52	52%
Asthma				4	4%
COPD				4	4%
Creatine level (> 1,2 mg/dl)				12	12%

BMI=body mass index; COPD=chronic obstructive pulmonary disease; SS=standard deviation values

All patients were evaluated based on KDIGO criterion, and AKI development was detected in 24 patients. AKI staging was performed in the AKI (+) patient group, and 18 patients (75%) were stage 1, two patients (8%) were stage 2, and four patients (17%) were stage 3.

RRT was needed in three cases after the operation, and two of these patients died within 30 days.

The perioperative characteristics of both AKI (+) and AKI (-) cases are shown in Table 2. No statistically significant difference was found between AKI (+) and AKI (-) groups in terms of age, gender, body mass index (BMI), and comorbidities such as hypertension and diabetes, however, risk of AKI development was found to be higher only in patients with a creatinine level > 1.2 mg/dl in the preoperative period. Surgeries being elective or emergency had no contribution to AKI development. When surgery types were examined, it was found that 50 patients underwent coronary artery bypass grafting (CABG), 15 patients underwent single valve replacement, seven patients underwent double valve replacement, seven patients underwent valve replacement + CABG, and 21 patients underwent left ventricular assist device (LVAD) surgery. No correlation was found between these groups in terms of AKI development.

**Table 2:** Characteristics of AKI (+) and AKI (-) groups.

		AKI (+)	AKI (-)	*P*-value		Total
		N=24	N=76			N=100
Age		65.76±7.7	60.58±2.64		>0.05	100
Gender	Female	4	25	>0.05		29
	Male	20	51	>0.05		71
Weight (kg)		76.25±2,25	77.7±1.5	>0.05		100
BMI (kg/m^2^)		26.11±0.11	27.4±0.5	>0.05		100
Hypertension	Existing	14	38	>0.05		52
	None	10	38			48
Diabetes mellitus	Existing	8	21	>0.05		29
	None	16	55			71
Asthma	Existing	0	4	>0.05		4
	None	24	72			96
COPD	Existing	1	3	>0.05		4
	None	23	73			96
Creatine level (> 1,2 mg/dl)	Existing	9	3	<0.05*		12
	None	15	73			88
OPAQ** duration (day)		32±7.7	25.9±2.7	<0.05		100
EuroSCORE		3.3±0.4	3.1±0.15	>0.05		100
Surgical planning	Elective	19	65	>0.05		84
	Emergent	5	11			16
Surgery type	CABG	13	37			50
	Single valve replacement	2	13			15
	Double valve replacement	2	5	>0.05		7
	CABG+valve replacement	3	4			7
	LVAD	4	17			21
Extubation period (hour)		24.7±6.2	10.4±1.8	<0.05*		100
Complication	Wound infection	0	5			5
	Atrial fibrillation	1	0			1
	Cardiac tamponade	7	1	<0.05*		8
	Pneumonia	3	2			5
	None	13	68			81

AKI=acute kidney injury; BMI=body mass index; CABG=coronary artery bypass grafting; COPD=chronic obstructive pulmonary disease; LVAD=left ventricular assist device

* Significant value.

** OPAQ is a contrast solution given to patients during angiography.

Among the haemodynamic data of patients measured in the intraoperative period, a significant difference was found in mean arterial pressure (MAP), central venous pressure (CVP), and pulmonary artery pressure (PAP); a difference between diastolic blood pressure and CVP (diastole-CVP) and a difference between MAP and CVP (MAP-CVP) were also found between AKI (+) and AKI (-) groups. There was no difference in terms of AKI development at the points where these parameters were measured. Table 3 shows haemodynamic data of AKI (+) patients and Table 4 shows haemodynamic data of AKI (-) patients.

**Table 3:** Hemodynamic data of AKI (+) patients.

	MAP	CVP	PAP	Diastole-CVP	MAP-CVP
Preinduction	79.41±2.02				
Postintubation	73.70±2.35	9.29±0.93	30.83±2.04	49.08±1.93	63±2.58
CPB inlet	72±2.40	7.04±0.86	29.70±2.14	48.58±2.13	63.45±2.28
CPB separation	71±3.17	8.62±0.78	29.33±1.68	47.16±3.71	60.91±3.07
End of surgery	76.41±2.77	8.79±0.80	27.87±1.89	50.83±2.71	65.33±3.72

AKI=acute kidney injury; CPB=cardiopulmonary bypass; CVP=central venous pressure; MAP=mean arterial pressure; PAP=pulmonary artery pressure

**Table 4:** Hemodynamic data of AKI (-) patients.

	MAP	CVP	PAP	Diastole-CVP	MAP-CVP
Preinduction	78.42±1.27				
Postintubation	73.53±1.73	9,18±0.46	26.47±1.07	49.80±1.34	66.42±1.65
CPB inlet	74.96±1.45	7.18±0.41	25.98±0.94	51.85±1.07	69.30±1.48
CPB separation	71.05±1.39	7.86±0.40	25.68±0.90	48.64±1.30	66.89±1.36
End of surgery	74.89±1.14	8.17±0.36	25.11±0.88	50.06±1.14	65.92±1.09

AKI=acute kidney injury; CPB=cardiopulmonary bypass; CVP=central venous pressure; MAP=mean arterial pressure; PAP=pulmonary artery pressure

Pump duration was 133±12 minutes in the AKI (+) group and 107.8±5.3 minutes in the AKI (-) group (p=0.03). There was no significant difference in cross-clamp time between the two groups (p=0.44). Cross-clamp time was 69.2±7.9 minutes in the AKI (+) group and 61.5±5 minutes in the AKI (-) group.

When fluid treatment applied to the patients in the intraoperative period was evaluated, the mean total crystalloid amounts were 1238±193.3 ml in AKI (+) patients and 1027±80.83 ml in AKI (-) patients (p=0.24). AKI (+) patients were treated with an average of 2.875±0.3146 units of erythrocyte suspension, and this was calculated as 1.592±0.1749 units in AKI (-) patients. Mean erythrocyte suspension was significantly higher in AKI (+) patients (*P*=0.0005). When fresh frozen plasma (FFP) units administered to patients in the intraoperative period were compared, it was found that mean FFP units administered to AKI (+) patients was 3.0±0.2692 units and that mean FFP units administered to AKI (-) patients was 2.197±0.1463 units. A significant difference was found between the two groups (*P*=0.009).

**Fig. 1:**
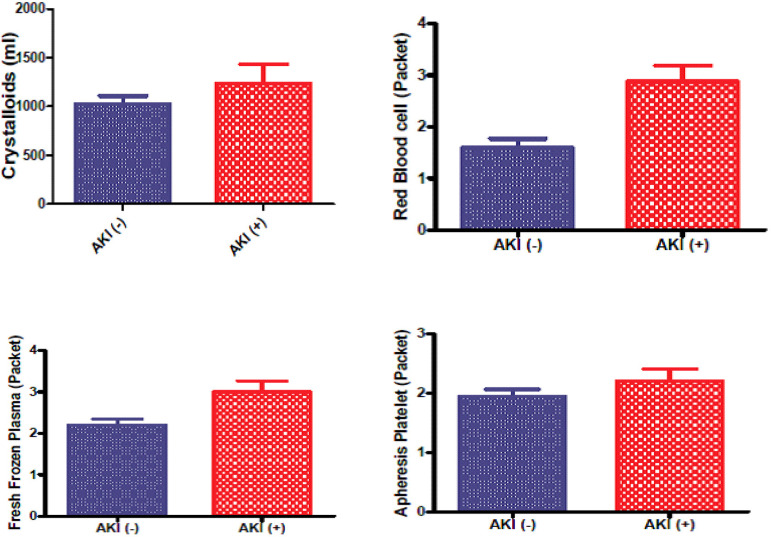
Intraoperative fluid therapy and transfusions. AKI=acute kidney injury

When total platelet suspension by apheresis administered to AKI (+) and AKI (-) groups in the intraoperative period were compared, it was found that the mean number of replacement units administered to AKI (+) patients was 2.208±0.1994 units and the mean number of replacement units administered to AKI (-) patients was 1.947 ± 0.1198 units (*P*=0.2814) (Figure 1).

When total urine volume in the intraoperative period was compared, mean total urine volume was 1223±133.6 ml in AKI (+) patients and 1467±92.79 ml in AKI (-) patients. No significant difference was found between the groups (*P*=0.1814).

The difference between systemic oxygen presentations (DO_2_) calculated between HLP activation and deactivation was investigated between the groups. DO_2_ measured after HLP activation was 413.1±15.47 ml/min/m^2^ in the AKI (+) group and 431.0±12.33 ml/min/m^2^ in the AKI (-) group (*P*=0.4505). DO_2_ measured after HLP deactivation was 411.4±16.39 ml/min/m^2^ in the AKI (+) group and 441.6±12.44 ml/min/m^2^ in the AKI (-) group (*P*=0.2115). No difference was observed between the two groups.

When intraoperative blood glucose changes were examined between the groups, it was found that blood glucose changes were higher in AKI (+) patients (*P*<0.05) than in AKI (-) patients.

When extubating times of all cases were examined, it was found that mean extubating time of AKI (+) patients was 24.74 ± 6.298 hours, whereas mean extubating time of AKI (-) patients was 10.49 ± 1.814 hours. Extubating time was longer in AKI (+) patients (*P*=0.0035).

When the patients were examined in terms of postoperative complications, the number of complications was significantly higher in AKI (+) patients (*P*=0.0004). Of the 24 AKI (+) patients, 13 had various postoperative complications. On the other hand, postoperative complications were observed in eight of 76 AKI (-) patients (Table 2).

In the analysis of candidate biomarkers for early detection of AKI, NGAL, IL-18, and cystatin C were studied in blood samples obtained in the preoperative period and the 3^rd^ postoperative hour.

**Fig. 2:**
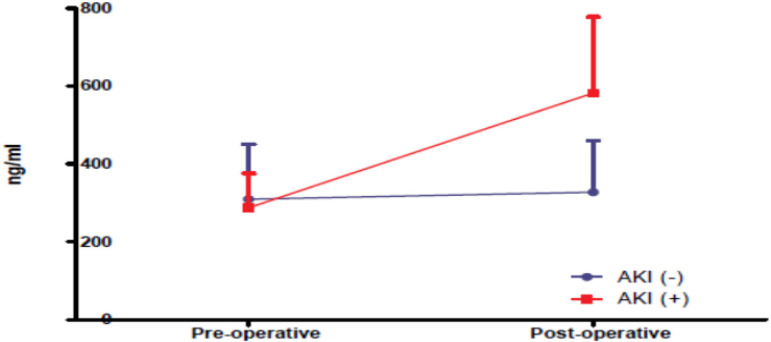
Neutrophil gelatinase-associated lipocalin changes in the 3. postoperative hour compared to the preoperative period. AKI=acute kidney injury

The mean preoperative level of NGAL was 287.6 ng/ml in AKI (+) patients, whereas the mean level measured at the 3^rd^ postoperative hour was 582.6 ng/dl. In AKI (-) patients, mean preoperative level of NGAL was 309.4 ng/dl and mean level measured at the 3. postoperative hour was 327.9 ng/dl. A significant increase of over 100% was observed in AKI (+) patients (P<0.001), whereas no significant difference was found in AKI (-) patients (Figure 2).

**Fig. 3:**
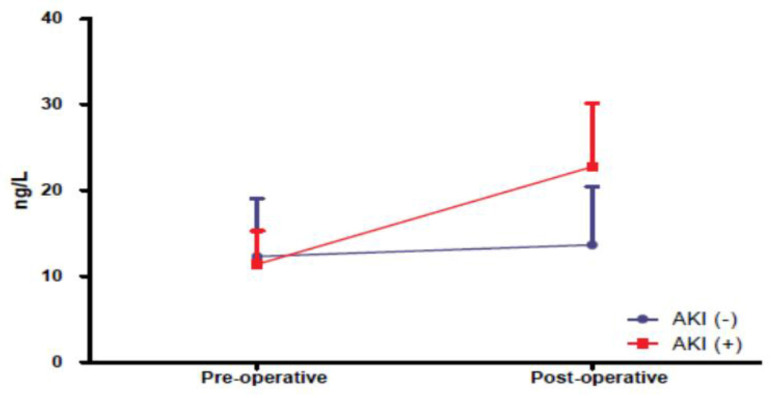
Interleukin-18 changes in the 3. postoperative hour compared to the preoperative period. AKI=acute kidney injury

The mean preoperative level of IL-18 was 11.41 ng/L in the AKI (+) group and the mean IL-18 level measured at the 3. postoperative hour was 22.75 ng/L. In the AKI (-) group, mean preoperative level of IL-18 was 12.33 ng/L and mean level measured at the 3. postoperative hour was 13.68 ng/L. There was a near 100% increase in IL-18 level in the AKI (+) group. A statistically significant difference was found between the two groups (*P*<0.001) (Figure 3).

**Fig. 4:**
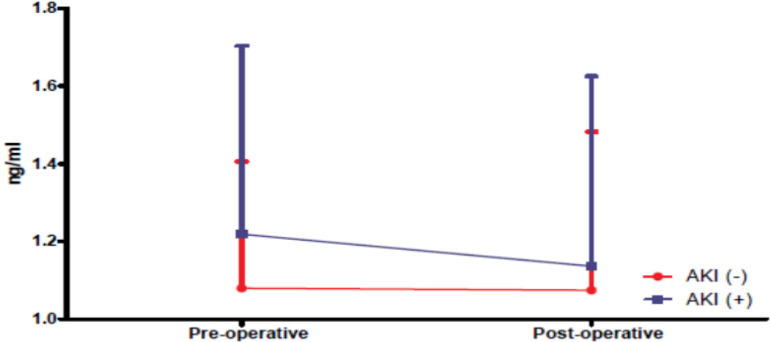
Cystatin C changes in the 3. postoperative hour compared to the preoperative period. AKI=acute kidney injury

The mean preoperative level of cystatin C was 1.219 ng/dl in the AKI (+) group and the mean cystatin C level measured at the 3^rd^ postoperative hour was 1.136 ng/dl. In the AKI (-) group, mean preoperative level of cystatin C was 1.080 ng/dl and mean level measured at the 3. postoperative hour was 1.075 ng/dl. No statistically significant difference was found between the two groups (*P*>0.05) (Figure 4).

## DISCUSSION

Although the incidence of AKI varies according to the identification criterion, the incidence based on the latest published clinical guideline for AKI, the KDIGO, varies between 3.1% and 42%. In a prospective study by Birnie et al. conducted in three centers and including 30,854 patients, the incidence of CSA-AKI ranged between 20.4% and 24.6%, and in patients with AKI, 17.4-13.7% of patients were stage 1, 2.2-2.8% of patients were stage 2, and 3.6-6% of patients were stage 3.

In the present study investigating CSA-AKI incidence in 100 patients, 24 patients (24%) developed AKI. Among the AKI (+) cases, we found that 18 (18%) patients developed stage 1 AKI, two (2%) patients developed stage 2 AKI, and four (4%) patients developed stage 3 AKI. The results of our study were consistent with the data in the literature^[4]^.

Preoperative risk factors increasing the risk of CSA-AKI include female gender, presence of diabetes mellitus, peripheral arterial disease, chronic obstructive pulmonary disease, intra-aortic balloon pump use in the preoperative period, emergency surgery, and preoperative high creatinine levels^[5]^.

In the cohort study performed by Birnie et al., female gender, age > 75, BMI value > 30 kg/m^2^, preoperative diabetes mellitus, hypertension, pulmonary diseases, low ejection fraction, and emergency surgery operations were found to be risk factors for AKI development^[4]^.

In our patients, mean age was 65.7±7.7 years in the AKI (+) group and 60.5±2.6 years in the AKI (-) group and both groups were under the high risk age group reported by Birnie et al.^[4]^. In addition, mean BMI values were < 30 kg/m^2^ in both groups (*P*>0.05).

The interaction of surgical method and factors associated with the patient causes the reduction of renal function in these patients. Conventionally, renal function is evaluated by measurement of serum creatinine and urine output volume. More sensitive parameters were needed because of the limitations of urine output and serum creatinine levels in the detection of AKI, and cystatin C, which is thought to be a more sensitive marker of renal function, was used for the early detection of postoperative AKI. However, no clear evidence has been found on this topic yet^[6]^.

Although there are many biomarkers proposed in recent years for the detection of both CSA-AKI and AKI in general, the success rates of these biomarkers in AKI detection are controversial. Among all proposed biomarkers, NGAL and IL-18 are the ones which are reported to be more sensitive than others in detecting AKI. Patients with a preoperative creatinine level > 1.2 mg/dl are likely to have the highest risk of developing AKI due to reduced renal reserves^[7]^. It was shown that the rate of AKI requiring dialysis during the postoperative period increased by 10-20% in patients with preoperative creatinine levels > 2.0 mg/dl.

In the present study, AKI developed in the postoperative period in nine of 12 patients with preoperative creatinine levels > 1.2 mg/dl, and three of these patients required RRT in the postoperative period. These results were consistent with Birnie et al. (2.9%)^[4]^.

Emergency surgery is known to be a risk factor for AKI development^[5]^. In our study, however, there was no difference between the cases in terms of AKI risk.

It is known that the contribution of cardiac surgery type to AKI development varies, and this rate was found to be 2-5% and 6.1% in patients with isolated CABG by Grayson and Yamauchi et al. respectively, and reported to increase up to 30% in cases treated with valve replacement together with CABG^[8]^.

Although renal perfusion increases in the early period in patients undergoing LVAD, renal venous congestion and impaired renal function are observed in the first month after surgery due to increased diastolic pressure. AKI rates in patients undergoing LVAD surgery vary between 10-28%^[9]^.

In our cases, AKI developed in 13 patients (26%) undergoing CABG (n = 50), four patients (18%) undergoing valve replacement (n = 22), three patients (42%) undergoing valve replacement + CABG (n = 7), and four patients (23.5%) undergoing LVAD surgery (n = 21). However, no significant difference was found between surgery types in terms of AKI development risk, and this was attributed to the low number of cases in the present study.

One of the most important factors for the development of both CSA-AKI and AKI in general is the maintenance of hemodynamics to provide adequate renal perfusion. In a meta-analysis of Zhang et al. covering 30,000 patients, hypotension (central arterial pressure [CAP] < 55 mmHg) developing in the intraoperative period was shown to increase AKI development^[10]^. During cardiac surgery and/or when vasoplegia such as septic shock develops, it is recommended to maintain CAP at 60-65 mmHg for adequate renal perfusion^[10]^.

In the study of Redfors et al.^[11]^ showing that renal perfusion and oxygen presentation were improved by increasing CAP in cardiac surgery, the recommended CAP value for adequate renal perfusion was 73 mmHg. In our study, CAP was > 70 mmHg both in the AKI (+) and AKI (-) groups at each time point when hemodynamic data were measured and calculated in our cases (preoperative, post-intubation, HLP activation, HLP deactivation, and postoperative), which may have caused the lack of a statistically significant difference.

In cases where the difference between preoperative CAP and HLP CAP is > 26 mmHg, AKI risk is reported to be increased. Based on data obtained from observational cohort studies, it is also known that increasing CVP values in addition to CAP changes that are observed when the preoperative period is compared with the intraoperative period have adverse effects on renal perfusion and contribute to AKI development by creating relative hypotension^[12]^. These parameters called mean perfusion pressure (MPP) (CAP-CVP = MPP) and diastolic perfusion pressure (DPP) (diastolic blood pressure-CVP = DPP) were described by Saito et al.^[12]^ and investigated in 7,814 patients who underwent cardiac surgery, and it was found that these parameters were lower in the AKI group. In the present study, there was no significant difference between the groups in terms of preoperative CAP, intraoperative MAP, CVP, and PAP, and the calculated MPP and DPP parameters (Tables 3 and 4).

Pathophysiological changes associated with HLP, systemic inflammatory response, changes in renal vasomotor tonus, destruction of red blood cells, pigment nephropathy, loss of pulsatile flow form, and final organ damage due to activation of the complement and coagulation cascade are the causes of AKI development. Mangano et al. state that this duration being > 180 minutes is an independent risk factor for postoperative AKI^[13]^. In our study, it was observed that HLP duration was longer (133±12 minutes) in patients who developed AKI than those without AKI. In another study of 100 cases, cross-clamp time > 90 minutes was reported to be another AKI risk factor^[14]^. In our study, cross-clamp time was < 90 minutes in both groups and this was consistent with the literature.

It is reported that blood transfusions performed during cardiac surgery are another risk factor for AKI development. These effects increase as the amount of transfused blood/blood products increases^[15]^.

In our study, transfused erythrocyte suspension and FFP units were found to be higher in AKI (+) cases. Although fluid treatment during operation is a treatment regimen frequently used in patients with AKI risk to correct hypovolemia, to ensure hemodynamic stability, to prevent toxicity-induced tubular damage, and to ensure adequate urine output necessary for the removal of metabolic residues from the body, the use of more sensitive parameters for optimal fluid treatment is becoming widespread. It is known that organ dysfunction developing under excessive volume burden increases morbidity and mortality rates^[16]^.

The first clinical symptom occurring in patients with AKI is decreased urine output volume; therefore, this symptom has been included in diagnostic algorithms developed for the diagnosis of AKI^[3]^.

Oliguria occurring in surgeries that cause high inflammatory and neurohormonal activation, such as cardiac surgery, may not always be associated with kidney damage. Increased levels of antidiuretic hormone (ADH) in these patients may also cause oliguria^[17]^. In our study, we detected oliguria in AKI (+) cases but we did not measure ADH.

In the study of Abramov et al.^[18]^, the mean systemic oxygen supply required to prevent kidney injury during HLP use was reported as 262 ml/min/m^2^. In our study, oxygen supply after HLP activation was measured as 413.1±15.47 ml/min/m^2^ in the AKI (+) group and 431.0±12.33 ml/min/m^2^ in the AKI (-) group (*P*=0.4505). Oxygen supply after HLP deactivation was measured as 411.4±16.39 ml/min/m^2^ in the AKI (+) group and 441.6±12.44 ml/min/m^2^ in the AKI (-) group (*P*=0.2115). No difference was found between the two groups.

In an observational study conducted by Azevedo et al.^[19]^, a significant correlation was found between high blood glucose levels and AKI incidence in critically ill patients, and Mehta et al.^[19]^ showed that hyperglycemia and insulin resistance directly contributed to AKI development. When we compared the preoperative and postoperative blood glucose levels of our cases, we found that the increase in blood glucose level was significantly higher in the AKI (+) group than in the non-AKI group.

Neurological and renal complications leading to increased mortality and morbidity following cardiac surgery can be listed as prolonged intubation and mechanical ventilation duration for various reasons, infections in the sternotomy incision site, complications related with other infections, cardiac dysrhythmias, and unscheduled resurgical needs. These complications may occasionally occur together, or one complication may result in the occurrence of the other. In a study of Crawford et al.^[20]^ on 2,477 patients, the complication rate after cardiac surgery was 19.8%, the rate of unscheduled reoperation was 2.9%, and the rate of neurological complications ranged from 0% to 5%.

In our study, we detected more postoperative complications in patients with AKI. In addition, AKI (+) patients had a longer duration of leaving mechanical ventilation compared to AKI (-) patients, unscheduled reoperation rate was found to be 8% in all patients, and this rate was 29% in AKI (+) patients. While pulmonary complications were seen in 5% of all cases, they were seen in 12.5% of AKI (+) cases. On the other hand, while infective complication rate was 5% in all cases, these complications were not observed in AKI (+) patients. Aside from one AKI (+) patient developing atrial fibrillation in the postoperative period, none of our patients developed neurological complications.

In a meta-analysis of 1,219 patients, Parikh et al. reported that some of the markers used were associated with length of hospitalization, dialysis need, and in-hospital mortality in addition to AKI detection^[11]^. One of the most researched markers for this purpose is cystatin C and the data reported in the literature so far is contradictory. In a prospective study of 114 patients, Haase et al.^[21]^ found that cystatin C values measured during delivery to the postoperative intensive care unit compared to preoperative values were significantly higher in patients with AKI compared to patients without AKI.

In a study of 150 patients, Wald et al.^[22]^ reported that cystatin C levels measured at 2^nd^ postoperative hour were lower than preoperative levels in the AKI group. In our study, we did not find any significant difference between preoperative cystatin C levels and cystatin C levels measured at the 3^rd^ postoperative hour.

One of the most promising markers in detecting AKI is NGAL. In a meta-analysis of 244 studies conducted by Haase et al.^[23]^, NGAL was reported to be highly successful in the detection of postoperative AKI, but large variations were observed in NGAL values. The cutoff values of NGAL in the detection of AKI ranged from 50 ng/dl to 550 ng/dl in different studies. As a result of the meta-analysis of all these studies, a NGAL level > 100-270 ng/dl (median value: 170 ng/dl) was defined as the cutoff value for AKI diagnosis in adults. In a study on 1,219 patients undergoing open-heart surgery, Koyner et al.^[24]^ reported that plasma NGAL levels were more successful than urinary NGAL levels in detecting AKI, and the cutoff value for plasma NGAL level was determined to be 323 ng/dl.

The mean preoperative NGAL values in our cases were 287.6 ng/dl in patients with AKI and 309.4 ng/dl in patients without AKI. At the 3^rd^ postoperative hour, mean NGAL values were 582.6 ng/dl in patients with AKI and 327.9 ng/dl in patients without AKI. In our study, a statistically significant increase of more than 100% was observed between the preoperative and the 3^rd^ postoperative hour NGAL values in patients who developed AKI (*P*<0.001) (Figure 2). Our results were consistent with the literature in terms of increased NGAL in AKI (+) cases, but the values measured were above the threshold specified by Haase et al.^[23]^. Our data on the high NGAL values are similar to other studies in the literature detecting high NGAL levels. Different NGAL values obtained in the literature may also be due to the measurement technique applied.

In a study conducted by Blankenberg et al., it was stated that serum IL-18 concentration may be a marker of the risk of developing coronary events^[25]^.

In our study, we found a significant increase of 100% in IL-18 levels measured at the 3^rd^ postoperative hour in AKI (+) cases compared to preoperative values.

## CONCLUSION

In our study, there was no difference between the preoperative demographic data identified as risk factors for AKI. In our sample of 100 patients, no difference was found in terms of female gender and perioperative haemodynamic parameters, and the measured values were above the specified threshold values indicated for AKI development risk. Blood/blood product amounts administered to patients in the intraoperative period and extubating times were higher in patients who developed AKI than in those without AKI. Systemic oxygen presentations in both groups were higher in both groups of patients than the levels reported in other studies for increased risk of developing AKI.

Cystatin C, NGAL, and IL-18 parameters were measured preoperatively and at the 3. postoperative hour to assess their value in early detection of kidney injury. No statistically significant difference was found between cystatin C levels in terms of AKI development risk. A statistically significant difference was found between the preoperative and the 3. postoperative hour measurements of NGAL and IL-18 levels in patients with AKI. There are only a few studies in the literature evaluating all three markers together.

We believe that further studies are needed to test the accuracy of markers used for early-stage kidney injury in order to identify the causes of AKI and take the necessary preventive measures.

**Table:** 

Author's roles & responsibilities	
HM	Substantial contributions to the conception or design of the work; or the acquisition, analysis, or interpretation of data for the work
EG	Substantial contributions to the conception or design of the work; or the acquisition, analysis, or interpretation of data for the work; drafting the work or revising it critically for important intellectual content; final approval of the version to be published; agreement to be accountable for all aspects of the work in ensuring that questions related to the accuracy or integrity of any part of the work are appropriately investigated and resolved
TAT	Substantial contributions to the conception or design of the work; or the acquisition, analysis, or interpretation of data for the work
IOK	Agreement to be accountable for all aspects of the work in ensuring that questions related to the accuracy or integrity of any part of the work are appropriately investigated and resolved
